# Feeding the world's largest fish: highly variable whale shark residency patterns at a provisioning site in the Philippines

**DOI:** 10.1098/rsos.170394

**Published:** 2017-09-27

**Authors:** Jordan A. Thomson, Gonzalo Araujo, Jessica Labaja, Emer McCoy, Ryan Murray, Alessandro Ponzo

**Affiliations:** 1 Large Marine Vertebrates Research Institute Philippines, Jagna, Bohol, Philippines; 2 Centre for Integrative Ecology, School of Life and Environmental Sciences, Deakin University, Warrnambool, Victoria 3280, Australia

**Keywords:** wildlife tourism, supplemental feeding, shark tourism, shark diving, habitat use, elasmobranch

## Abstract

Provisioning wildlife for tourism is a controversial yet widespread practice. We analysed the residency patterns of juvenile whale sharks (*Rhincodon typus*) in Oslob, Philippines, where provisioning has facilitated a large shark-watching operation since 2011. We identified 208 individual sharks over three years, with an average of 18.6 (s.d. = 7.8, range = 6–43) individuals sighted per week. Weekly shark abundance varied seasonally and peak-season abundance (approx. May–November) increased across years. Whale sharks displayed diverse individual site visitation patterns ranging from a single visit to sporadic visits, seasonal residency and year-round residency. Nine individuals became year-round residents, which represents a clear response to provisioning. The timing of the seasonal peak at Oslob did not align with known non-provisioned seasonal aggregations elsewhere in the Philippines, which could suggest that seasonal residents at Oslob exploit this food source when prey availability at alternative sites is low. Since prolonged residency equates to less time foraging naturally, provisioning could influence foraging success, alter distributions and lead to dependency in later life stages. Such impacts must be carefully weighed against the benefits of provisioning (i.e. tourism revenue in a remote community) to facilitate informed management decisions.

## 1. Background

Providing animals with food to increase or enhance sightings in wildlife tourism is a controversial practice. This is, in part, because the impacts on animal behaviour and welfare are often not well understood [1]. This knowledge gap has contributed to diverse opinions on the ethics and sustainability of provisioning and a similarly diverse set of permitted provisioning practices [2]. Of particular concern are possible negative impacts of provisioning on animal behaviour and their long-term consequences. For example, provisioning has been shown to increase intra-specific aggression [3,4], alter movement patterns [5,6] and activity profiles [3,7], reduce offspring survivorship [8] and parental care [9], and may disrupt foraging during non-provisioned periods [10]. Furthermore, provisioning can have physiological costs including reduced body condition [4], increased stress levels [11] and altered disease transmission dynamics [12]. While not all effects of provisioning are likely to be negative—indeed, animals can also benefit from supplemental feeding [1,13]—the various costs and benefits of this activity must be carefully weighed when making management decisions.

Elasmobranchs feature prominently in the provisioning debate because shark viewing is a rapidly growing, global tourism industry with annual revenues conservatively estimated at US $314 million [14,15]. Tour operators in many locations use food or attractants to aggregate sharks and rays to increase the reliability of sightings [16]. However, only recently have the effects of provisioning on elasmobranch behaviour begun to receive empirical attention [16,17]. The results of this research have been mixed. For example, some studies have found shifts in shark activity or movement patterns in response to provisioning [5,18,19] while others have found no substantial change in behaviour [20–22]. In general, the emerging body of research on shark provisioning suggests that its impacts are likely to be species- and site-specific, which makes a generalized management framework challenging [16].

Whale sharks (*Rhincodon typus*) are a major focus of the shark-viewing tourism industry [23], with approximately one-third of commercial shark-viewing operations advertising opportunities to view this iconic species [15] and revenues at one site (Maldives) estimated at approximately US $8 million annually [24]. This industry is facilitated by predictable seasonal peaks in whale shark sightings, which typically last from one to a few months, at many sites around the world [25]. The drivers of these aggregations are not yet well understood but are thought to include fluctuations in sea surface temperature, food blooms, and oceanographic features such as depth and upwelling [25–27]. However, whale shark movements can be complex, with some individuals making long-distance migrations of hundreds or thousands of kilometres and others remaining relatively close to these aggregation sites [25].

Nearly all whale shark-viewing operations rely on opportunistic sightings of non-provisioned animals. Therefore, concerns surrounding the impacts of tourism on whale sharks mostly pertain to disturbance caused by boats and people in the water [28–30]. However, at a globally unique site in the Philippines (Oslob, Cebu), a large tourism operation has emerged since 2011 around the provisioning of juvenile whale sharks by local fishermen in a small area (0.065 km^2^) at a highly accessible coastal location [31]. This year-round food source represents a major deviation from natural patterns of prey availability for a highly mobile planktivore. While whale sharks were sighted sporadically at Oslob prior to the start of provisioning in 2011 (E. Fernandez-Benologa 2012, personal communication), predictable daily sightings of large numbers of sharks have only occurred since provisioning began. Shark-viewing operations in Oslob hosted a minimum of 182 000 tourists in 2015 (unpublished tourist logbook data 2016, Local Government Unit, Oslob), creating the potential for significant impacts on the sharks (e.g. disturbance). Since whale sharks are listed as Endangered and the Indo-Pacific subpopulation has declined by an estimated 63% over the past three generations [32], it is critical to evaluate how provisioning may influence this population.

Araujo *et al*. [31] conducted the first investigation of the impacts of provisioning on whale shark behaviour at Oslob. Using photo-ID, they found that whale sharks that were observed being hand-fed had a longer mean residence time (45 days) than sharks that visited the site but were not observed being hand-fed (22 days). Thus, provisioning appears to alter whale shark movement patterns on the scale of weeks to months in this region. Here, we extend and expand on this work. Specifically, we: (1) assess the number of individual sharks visiting the provisioning site per week over three years; (2) use sightings frequency thresholds and co-occurrence analysis to elucidate residency patterns within this aggregation; and (3) qualitatively interpret these residency patterns in relation to known whale shark movements at non-provisioned sites in the Philippines and elsewhere in order to assess the impact of provisioning on shark behaviour. Our overarching goal is to inform the debate surrounding whale shark provisioning in the Philippines while enhancing our understanding of shark movement ecology and wildlife provisioning impacts in general.

## 2. Material and methods

### 2.1. Field surveys

**Figure 1.**
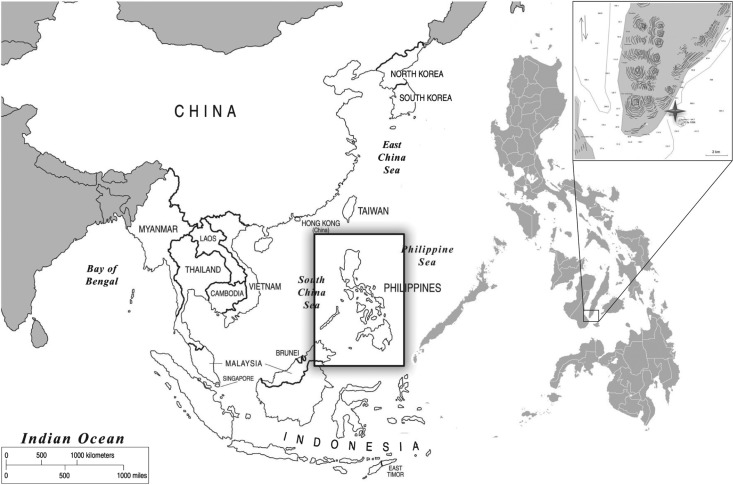
Map showing the Philippines and the location of the study site in Oslob at the southern end of Cebu Island (inset).

**Figure 2.**
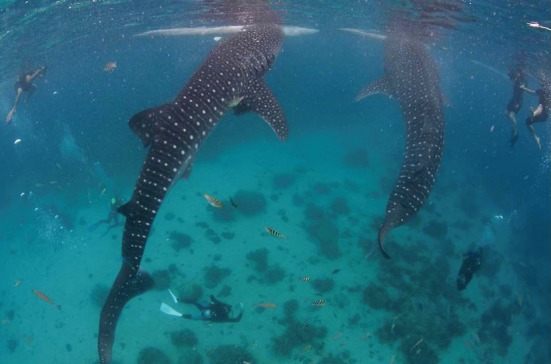
Photograph showing two whale sharks feeding vertically from feeder boats at Tan-Awan while snorkelers and SCUBA divers look on. Photo credit: Steve De Neef.

The municipality of Oslob is located at the southern end of Cebu Island, Philippines (figure 1). Within a small (0.065 km^2^), demarcated interaction area located 50–100 m from shore at the village of Tan-Awan, whale sharks are hand-fed krill continually from 06.00 to 13.00 h every day by feeders operating one-man paddleboats. Larger paddleboats bring groups of guests out for surface-based or in-water shark viewing while SCUBA divers can enter the site from shore or motorized boats moored at the edge of the interaction area (figure 2).

From 31 March 2012 to 31 March 2015, trained observers conducted photo-ID surveys of whale sharks in the interaction area throughout the daily provisioning period (see [31]). In 1-h shifts, one or more observers searched the interaction area haphazardly while snorkelling, focusing predominantly on areas where feeder boats were concentrated but also making occasional sweeps of the entire demarcated area. When a shark was encountered, its left flank was photographed between the pectoral and dorsal fins, level with the dorso-ventral midline [33]. Genital, right flank and other opportunistic photos (e.g. scars) were then obtained for sharks that were not known or previously well photographed. For new sharks (i.e. those not previously identified), sex was determined based on the presence of claspers and size estimates were made using visual estimation or laser photogrammetry (see [31]). Photos were then matched manually against a digital catalogue to create a daily shark presence–absence database showing the site visitation history of each individual. For approximately the first two years of the study (31 March 2012 to 20 February 2014), surveys were conducted from 07.00 to 08.00 h, 09.00 to 10.00 h and 11.00 to 12.00 h daily. After 21 February 2014, the observer programme was expanded and surveys were conducted continuously from 06.00 to 13.00 h, still in 1-h shifts. We tested for the effect of this methodological change when analysing trends in weekly shark abundance (see Weekly shark abundance).

### 2.2. Data analysis

#### 2.2.1. Weekly shark abundance

Statistical analyses were performed in R v. 3.0.2 [34]. First, we used a generalized linear regression model (GLM) with Poisson errors to examine variation in the number of unique individual sharks sighted per week at the provisioning site over three years. We began with a maximal model that included: (1) a time variable (*t*, weeks since the beginning of the study) to test for an overall trend; (2) sine and cosine components to test for seasonality [35]; (3) an interaction between *t* and the seasonal components to test for variation in the amplitude of the seasonal cycle; and (4) weekly survey effort (i.e. number of hours with at least one observer in the water). We could not adjust weekly counts for survey effort prior to modelling because the doubling of survey effort after 21 February 2014 resulted in very few additional sharks being identified and, therefore, resulted in extremely low effort-adjusted counts compared with previous years. Instead, we used the model to test whether variation in survey effort had a significant effect on the number of individual sharks sighted per week. Unfortunately, we lacked data to test the effect of variation in the amount of food provided or number of boats and people in the interaction area on shark abundance. We attempted to simplify the maximal model by removing individual terms (sine and cosine terms were removed together), starting with the highest-order terms, and comparing the fit of nested models using analysis of deviance [36]. The final model was validated using diagnostic plots and by calculating a pseudo-*R*^2^ value, which estimated the proportion of variance in the number of sharks sighted per week explained by the model [36].

#### 2.2.2. Residency patterns

To elucidate temporal patterns of provisioning site visitation, we analysed shark co-occurrence using permutation tests and principal coordinate analysis (PCoA) following the method developed by Clua *et al*. [37]. This allowed us to categorize whale shark residency patterns based on among-individual similarities in daily presence–absence histories. However, due to the large number of whale sharks identified in our study (*n* = 208), and strong among-individual variation in dates of initial identification and frequencies of occurrence, it was not possible to assess co-occurrence of all sharks in a single analysis. To address this, we took the following steps. First, we classified sharks based on the year of the study in which they were identified (Y1 = 31 March 2012–30 March 2013; Y2 = 31 March 2013–30 March 2014; Y3 = 31 March 2014– 31 March 2015). Second, we used frequency-of-sighting thresholds to divide all sharks into three groups: single-sighting, infrequent and frequent sharks. Single-sighting sharks were those that were photographed once and confirmed to be unique individuals but were not re-sighted during the study. Infrequent sharks were those that were seen on less than 30 days, on average, per year known (i.e. less than 90 days for Y1 ID sharks, less than 60 days for Y2 ID sharks and less than 30 days for Y3 ID sharks). This threshold was determined based on preliminary permutation test and PCoA trials, which revealed that sharks not meeting this threshold had few significant associations, thus precluding further grouping of their residency patterns. Frequent sharks were those that were sighted on at least 30 days, on average, per year known. Co-occurrence analysis was conducted on frequent sharks only. To avoid assessing co-occurrence between frequent sharks that could not have overlapped for substantial lengths of time, we analysed individuals identified in each study year separately (hereafter Y1 ID, Y2 ID or Y3 ID). Note that we analysed co-occurrence from the year of ID until the end of the study as opposed to from year to year. Together, these steps ensured that we avoided generating overly complex PCoA plots with many individuals but few significant associations.

We constructed date-by-shark matrices for Y1 ID, Y2 ID and Y3 ID frequent sharks showing the daily presence–absence history of each individual. These matrices were then converted into dissimilarity matrices showing the number of co-occurrences for all pairs of individuals within each analysis. This statistic is usually denoted as *a* in descriptions of binary similarity indices [38]. We then used the R function of Clua *et al*. [37] to carry out tests of *a* by permutation following the method originally proposed by McCoy *et al*. [39] and detailed by Legendre & Legendre [38]. We performed 9999 random permutations to obtain a matrix of *p*-values associated with the *a* statistics, testing against the null hypothesis of no association between a given pair of sharks. We used Holm correction to account for multiple comparisons. *p*-values < 0.05 following Holm correction indicated a significant association between a pair of individuals.

The matrices of *p*-values were used to categorize frequent shark residency behaviour at the provisioning site. As in Clua *et al*. [37], agglomerative clustering was not useful for this problem because groups of sharks were not clearly isolated from one another. We therefore used PCoA of the matrices of original *p*-values to group sharks based on significant associations [38]. In order to avoid negative eigenvalues resulting from non-Euclidean distances in the matrices, a constant was added to each dissimilarity value [38,40]. Sharks were plotted as individual points on a PCoA plot and lines were drawn between points for individuals with significant associations. Residency groups were determined via visual inspection of these plots. To provide an indication of model fit, we calculated an *R*^2^-like value following Legendre & Legendre [38], which estimated the amount of variation in each dissimilarity matrix explained by the first two PCoA axes.

#### 2.2.3. Group-level daily abundance

To assess how the abundance of sharks with different residency patterns varied over time, we plotted the number of sharks sighted per week in four residency groups: single-sighting, infrequent and two categories of frequent sharks (see Shark residency patterns). We did not attempt to fit statistical models at the group level due to highly nonlinear patterns for some groups (see Group-level weekly abundance) and the limited value of fitting complex models to short-term time series data without a strong basis for biological interpretation. Rather, these plots were examined visually.

#### 2.2.4. Size and sex effects

In many elasmobranchs, including whale sharks [41], size and sex are known to influence broad-scale movements [42,43]. As in previous research at Oslob [31], we used a Pearson's *χ*^2^ test to confirm male bias in this aggregation, which is consistent with most Indian Ocean whale shark aggregations [25]. We then compared the mean size (at the time of initial estimation) of the four residency groups using ANOVA with post hoc Tukey's HSD tests. For sex, the relatively small number of females precluded quantitative analysis at the group level so these data were examined visually for patterns that might warrant additional investigation in the future.

## 3. Results

### 3.1. Data summary

In total, 208 individual whale sharks were identified at the provisioning site in 4710 h of survey effort during the three-year study. The mean size of sharks for which estimates were available (*n* = 194) was 5.3 m (s.d. = 1.4, range = 2.5–9.0). Sex classification was possible for 197 sharks and the aggregation was significantly male-biased (*χ*^2^ = 47.5, *p* < 0.001), with 164 males and 33 females identified (see also [31]).

### 3.2. Weekly shark abundance

**Figure 3.**
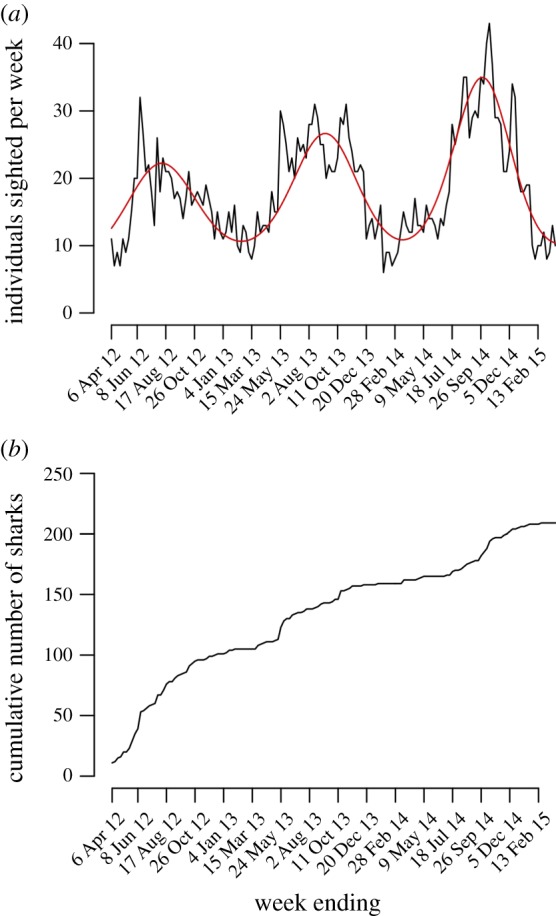
(*a*) Number of unique individual whale sharks (black line) sighted per week at the provisioning site between 31 March 2012 and 31 March 2015. The red line is the fitted curve from the GLM. (*b*) Discovery curve showing the cumulative total number of sharks identified since the beginning of the study.

An average of 18.6 individual sharks (s.d. = 7.8, range = 6–43) were sighted per week at the provisioning site over the course of the study. Weekly shark abundance showed a positive trend over time as well as a pronounced seasonal cycle, with the seasonal amplitude increasing over three years (figure 3*a*, table 1). The final model explained 71.6% of variation in the number of individual sharks sighted per week. Survey effort had no effect on the number of sharks seen per week (*L* = 2.041, d.f. = 1, *p* = 0.153). Seasonal peaks in shark abundance generally occurred between May and November each year. A maximum of 31 individual sharks were sighted at the provisioning site on a single day, for two consecutive days, in October 2014. The cumulative number of sharks identified at the site increased steadily from year to year, with several periods of rapid influx of newly identified individuals corresponding with the first few months of the study and subsequent peak seasons (figure 3*b*).

**Table 1.** Results of the final GLM investigating temporal variation in weekly whale shark abundance at the provisioning site in Oslob over three years.

parameter	estimate	s.e.	*t*	*p*
intercept	2.664	0.042	62.809	<0.001
*t*	0.002	0.000	5.127	<0.001
sin(*t*)	0.370	0.059	6.280	<0.001
cos(*t*)	−0.175	0.056	−3.128	0.002
*t* × sin(*t*)	−0.003	0.001	−4.474	<0.001
*t* × cos(*t*)	−0.003	0.001	−5.384	<0.001

### 3.3. Shark residency patterns

Whale sharks at Oslob displayed a diverse array of provisioning site visitation patterns ranging from a single visit to multiple sporadic visits, seasonal residency and year-round residency (electronic supplementary material, figure S1). Among Y1 ID sharks (*n* = 108), 21 (19%) were single-sighting, 67 (62%) were infrequent and 20 (19%) were frequent sharks. Among Y2 ID sharks (*n* = 53), 14 (26%) were single-sighting, 31 (58%) were infrequent and 8 (15%) were frequent sharks. Among Y3 ID sharks (*n* = 47), seven (15%) were single-sighting, 32 (68%) were infrequent and 8 (17%) were frequent sharks (table 2).

**Table 2.** Summary of sightings frequency data for 208 whale sharks identified photographically at a provisioning site in Oslob, Philippines between 31 March 2012 and 31 March 2015. Infrequent sharks were sighted on less than 30 days per year known on average while frequent sharks were sighted on at least 30 days per year known on average. Statistics presented for sightings individual^−1^ are the mean (s.d.) and range.

year of ID	frequency group	no. sharks	sightings individual^−1^
Y1 (*n* = 108)	single-sighting	21 (19%)	1
	infrequent	67 (62%)	17.0 (16.8)
			2–87
	frequent	20 (19%)	478.7 (261.7)
			103–887
Y2 (*n* = 53)	single-sighting	14 (26%)	1
	infrequent	31 (58%)	12.6 (12.1)
			2–45
	frequent	8 (15%)	139.8 (74.7)
			67–248
Y3 (*n* = 47)	single-sighting	7 (15%)	1
	infrequent	32 (68%)	9.6 (6.4)
			2–27
	frequent	8 (17%)	78.8 (44.1)
			37–166

**Figure 4.**
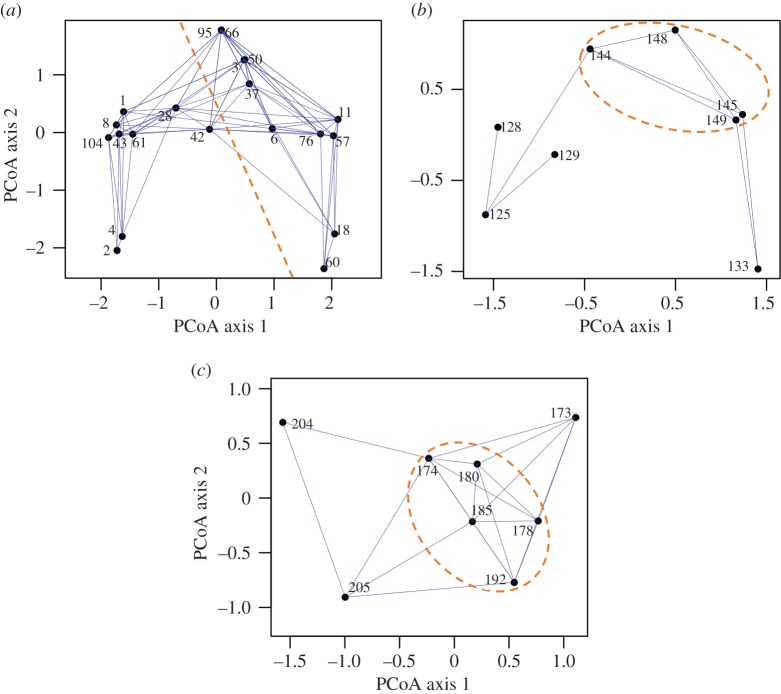
PCoA plot showing significant associations and group membership in Y1 ID frequent sharks (*a*), Y2 ID frequent sharks (*b*) and Y3 ID frequent sharks (*c*). For Y1 ID frequent sharks (*a*), highly resident sharks are located to the left of the orange, hashed line while seasonal sharks are located to the right of the line. For Y2 ID and Y3 ID sharks (*b*,*c*), the orange, hashed ellipses contain seasonal sharks.

**Figure 5.**
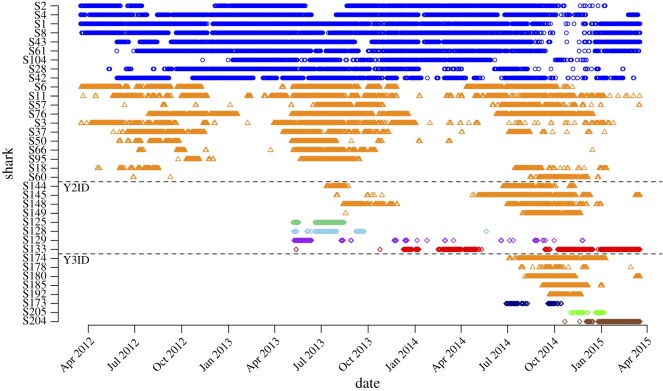
Daily attendance histories at the provisioning site for Y1 ID, Y2 ID and Y3 ID frequent sharks. Colours and symbols indicate groups of sharks identified based on visual examination of PCoA plots (blue circles, highly resident sharks; orange triangles, seasonal sharks; diamonds/other colours, frequent sharks that did not fall in one of these groups).

The PCoA plot for Y1 ID frequent sharks suggested that they could be further divided into two residency groups: highly resident and seasonal sharks (figures 4*a* and 5). Highly resident sharks (*n* = 9, to the left of the orange dashed line in figure 4*a*, blue symbols above the Y2 ID line in figure 5) were present at the provisioning site year round with no evidence of seasonal movements and few prolonged absences. Within this group, subtle variation could be discerned. Sharks S2 and S4 were present nearly daily throughout the study with the exception of two extended (greater than 1 month), overlapping absences including one in the final months of the study. These individuals clustered together and only had significant associations with other highly resident sharks. Sharks S1, S8, S43, S61 and S104 were present consistently from the date of initial identification to the end of the study but most shared a common absence spanning several weeks in the final months of 2014. These sharks primarily had significant associations with other highly resident sharks but also had some significant associations with seasonal sharks. Sharks S28 and S42 were present at the provisioning site throughout the three-year study but had several short, irregular absences and a reduced frequency of sightings in the final six months of the study. These individuals shared many significant associations with both highly resident and seasonal sharks.

Y1 ID seasonal sharks (*n* = 11, to the right of the orange dashed line in figure 4*a*, orange symbols above the Y2 ID line in figure 5) tended to be present at the provisioning site between approximately May and November, though not necessarily in all years (figure 5). Two individuals (S18, S60) displayed a bookend pattern, with sightings occurring in the first and third peak seasons of the study but not the second. These individuals clustered together and had significant associations almost exclusively with other seasonal sharks. Several seasonal sharks (e.g. S6, S11, S57, S76) showed a strong pattern with near-daily sightings at the provisioning site for 5–7 months in each year (although S57 was seen only irregularly during Y1 peak season). These sharks shared significant associations with other seasonal sharks and, rarely, with highly resident sharks. The remaining seasonal sharks (S3, S37, S50, S66, S95) were seen relatively frequently during Y1 and Y2 peak season but were seen rarely, if at all, during Y3. These sharks had significant associations with many other seasonal and highly resident sharks. The first two PCoA axes explained 27.2% of the variance in the Y1 ID frequent shark dissimilarity matrix.

The PCoA plot for Y2 ID frequent sharks revealed one clear cluster comprising four sharks showing evidence of seasonal residency (i.e. presence in two consecutive years during peak season, although one individual, S149, was seen only once during Y2). These four individuals had significant associations with each of the other sharks in the cluster. Sharks S125 and S128 were sighted regularly for approximately three months following initial identification at the beginning of Y2 peak season but were seen rarely or not at all for the remainder of the study. Shark S129 was sighted regularly for approximately one month following initial identification at the beginning of Y2 peak season and was then seen sporadically throughout the remainder of the study. Shark S133 adopted a seasonal residency pattern whose timing did not align with other seasonal sharks (i.e. June–November). Instead, S133 was present at the provisioning site regularly between January and April in Y2 and Y3. The first two PCoA axes explained 58.2% of the variance in the Y2 ID frequent shark dissimilarity matrix.

The PCoA for Y3 ID frequent sharks revealed one clear cluster of five individuals that were regularly present at the provisioning site during Y3 peak season (contained by the orange ellipse in figure 4*c*; orange symbols below the Y3 ID line in figure 5). Two other individuals, S173 and S205, shared significant associations with several Y3 ID seasonal sharks but their residency patterns differed, comprising two short bouts of presence at either the beginning or end of Y3 peak season. We did not consider these individuals as seasonal sharks pending further data. Finally, shark S204 was initially identified in November 2014 and was present almost daily from January–April 2015, which appeared similar to the off-peak seasonal residency of S133 (Y2 ID). The first two PCoA axes explained 63.5% of the variance in the Y3 ID frequent shark dissimilarity matrix.

### 3.4. Group-level weekly abundance

**Figure 6.**
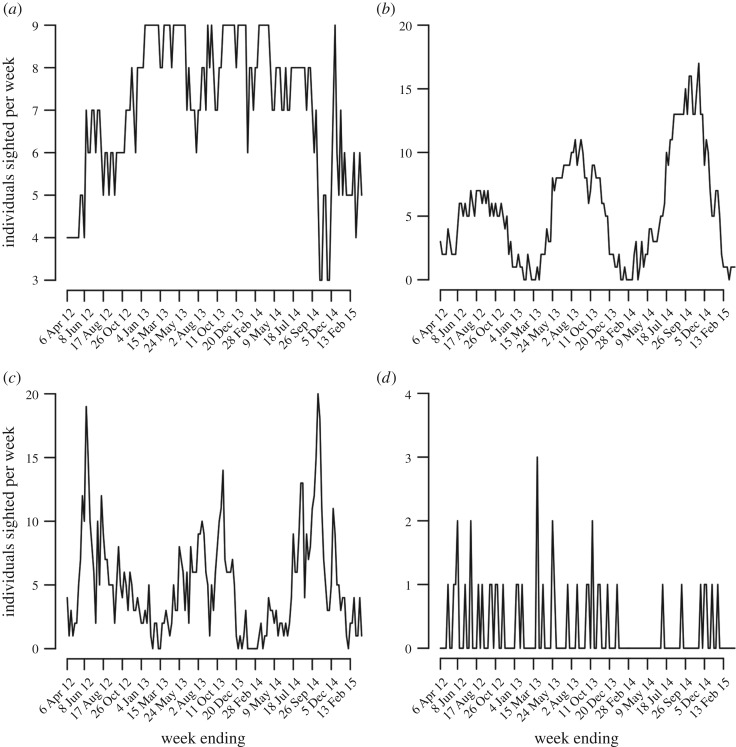
Weekly abundance at the provisioning site of four residency groups: highly resident sharks (*a*), seasonal sharks (*b*), infrequent sharks (*c*) and single-sighting sharks (*d*).

The four residency groups—single-sighting, infrequent, seasonal and highly resident sharks—showed very different patterns of abundance over time. The number of highly resident sharks sighted per week ranged from 3–9 (mean = 7.1, s.d. = 1.7) and varied nonlinearly over the course of the study, with a notable dip in late 2014 and subsequent partial recovery in early 2015 (figure 6*a*). The number of seasonal sharks sighted per week ranged from 0 to 17 (mean = 5.5, s.d. = 4.2), showed the expected strong seasonality, and increased during peak season in each year of the study (figure 6*b*). The number of infrequent sharks sighted per week ranged from 0–20 (mean = 4.9, s.d. = 4.0) and varied erratically from week-to-week but with spikes typically occurring during peak season (figure 6*c*). The number of single-sighting sharks sighted per week ranged from 0–3 (mean = 0.3, s.d. = 0.5) and showed no discernible pattern over time (figure 6*d*).

### 3.5. Size and sex effects

Shark size varied significantly among the four residency groups (*F*_3,184_ = 3.560, *p* = 0.015), although residency group explained a very low proportion of the variation in body size (*r*^2^ = 0.039). Highly resident sharks were the smallest followed by seasonal sharks, infrequent sharks and single-sighting sharks, although only the difference between single-sighting and highly resident sharks was significant (table 3). No clear differences in sex composition existed between groups, although highly resident and seasonal sharks had a higher proportion of females than other groups (table 3).

**Table 3.** Mean body size and sex composition of whale shark residency groups at the provisioning site in Oslob. For size estimates, standard errors are given in parentheses after the mean and superscripts show the results of Tukey's post hoc tests (groups not sharing a superscript letter are significantly different). The proportion of male sharks in each group is given in parentheses following the frequency.

residency group	*n*	number with size estimate	size (m)	number with known sex	no. male	no. female
highly resident	9	9	4.3 (0.4)^a^	9	7 (0.78)	2
seasonal	21	21	5.0 (0.3)^a,b^	21	13 (0.62)	8
infrequent	130	128	5.3 (0.1)^a,b^	130	112 (0.86)	18
single-sighting	42	30	5.8 (0.3)^b^	31	26 (0.84)	5

## 4. Discussion

If not carefully managed, marine wildlife tourism can negatively impact the populations upon which it depends [44,45]. Provisioning can have significant long-term impacts, both positive and negative, on individuals, populations and communities. For example, supplemental feeding of birds can increase reproductive output and drive range expansion by increasing survivorship. However, it can also lead to reliance on human feeding, exacerbate disease transmission and have negative knock-on impacts on bird prey via increased predation around provisioning sites [46]. For long-lived marine vertebrates, it can be challenging to evaluate the long-term impacts of provisioning. In a notable exception, bottlenose dolphin (*Tursiops* sp.) calves in Shark Bay, Western Australia, had higher mortality rates when born to provisioned versus non-provisioned mothers [8], possibly due to reduced parental care by provisioned mothers [9]. For highly mobile and difficult-to-observe whale sharks, it is extremely challenging to monitor and compare aspects of the fitness of individuals under provisioned and non-provisioned conditions. As such, we must instead evaluate short-term responses to provisioning, interpret them in the context of our best estimate of their likely long-term consequences, and follow the precautionary principle.

### 4.1. What are ‘natural’ whale shark residency patterns?

A controlled comparison of whale shark residency behaviour under provisioned and non-provisioned conditions was not possible in this case due to the lack of equivalent monitoring at a non-provisioned site. However, several comparisons can be made within and among whale shark aggregations to contextualize the patterns we observed at Oslob. First, Araujo *et al*. [31] estimated that whale sharks that were present but not hand-fed at Oslob had a mean residence time of 22.4 days (s.e. = 8.9) compared with 44.9 days (s.e. = 20.6) for those that were hand-fed. Second, whale sharks at southern Panaon Island, Southern Leyte (approx. 200 km northeast of Oslob), where provisioning does not occur, had a mean residence time of 27.0 days (s.e. = 8.5), similar to non-hand-fed sharks at Oslob [31,47]. As such, one month appears to be a reasonable estimate of the mean residence time of non-provisioned juvenile whale sharks at foraging sites in the southern Philippines. Studies of whale shark aggregations in other coastal regions have also estimated mean residence periods of approximately one month (e.g. Ningaloo, Australia [48]) or less (e.g. Utila, Honduras [49]). While there can be considerable variability around these means (e.g. one shark at Southern Leyte was identified 17 times over 108 days in 2013 [47]), there is little evidence of year-round residency by whale sharks in nearshore surface waters under non-provisioned conditions [25]. Therefore, the near-daily presence of highly resident sharks feeding at the surface in Oslob appears to be a clear behavioural response to provisioning.

Whether provisioning has influenced the movements of seasonal sharks at Oslob is more challenging to discern. Seasonality is a well-known feature of non-provisioned whale shark aggregations globally [25]. In the Philippines, whale sharks aggregate in Donsol Bay (Sorsogon), located approximately 370 km north of Oslob, annually between November and May [28]. This seasonal aggregation has sustained a shark-viewing tourism industry since 1997, receiving up to 30 000 tourists per season (WWF-Philippines 2016, unpublished data). Another aggregation occurs in Southern Leyte during the same months [47], and between 600 and 2000 tourists per year view whale sharks at this location [50]. Several sharks identified at Southern Leyte have been confirmed via photo-ID to visit other feeding sites including Oslob, Donsol and locations further afield (e.g. Taiwan [47]). Therefore, whale sharks in this region are known to move seasonally and use multiple areas for feeding in the absence of provisioning (see also [51]). However, because some seasonal sharks remained at the Oslob provisioning site for more than eight months at a time (figure 5), it seems likely that provisioning has increased residency in these individuals.

Interestingly, peak whale shark season at Oslob broadly corresponds with times of year when sharks are not present at the non-provisioned sites of Donsol and southern Leyte (i.e. June—October, figure 3). As such, it is possible that seasonal sharks at Oslob (*n* = 20, 10% of all sharks identified) exploit this supplemental food source during times when prey availability at alternative sites is low or patches are distributed sparsely. However, despite known connectivity among coastal aggregation sites in the region (via photo-ID), published tracking datasets are currently limited (but see [51]) and very little is known about the environmental drivers (e.g. sea surface temperature, productivity) of seasonal whale shark movements throughout the Coral Triangle [25]. As a result, such hypotheses cannot yet be tested. This is clearly a priority area for future research.

The majority of whale sharks at Oslob (*n* = 130, 63% of all sharks identified) were infrequent visitors to the provisioning site based on our criteria (less than 30 sightings, on average, per year known). The occurrence of these individuals increased during peak season (figure 6*c*), which suggests a seasonal influx of sharks into the waters near Oslob but only occasional forays to the provisioning site by these individuals. As such, it is unlikely that provisioning has significantly altered the long-term movements of these sharks. Single-sighting sharks (*n* = 42, 20% of all sharks identified) showed no pattern in the timing of occurrence (figure 6*d*), and these individuals did not return to the provisioning site during the study. As such, their long-term behaviour has not been meaningfully affected. The remaining individuals (*n* = 7, 3% of all sharks identified) were frequent sharks whose presence–absence histories did not clearly group with other sharks. Notably, two of these individuals (S133 and S204) appeared to adopt a prolonged seasonal residency pattern whose timing did not align with that of other seasonal sharks (figure 5). This suggests both the potential for behavioural modification as a result of provisioning and a different driver (or combination of drivers) underlying the movements of these individuals.

While changes in the horizontal movements of whale sharks in response to provisioning could underlie the aggregation at Oslob, a shift in vertical movements is an important and not mutually exclusive alternative to consider. Whale sharks have been sporadically reported near Oslob since before the start of provisioning in 2011 (E. Fernandez-Benologa 2012, personal communication). It is possible that more sharks were resident in the area prior to 2011 but they were rarely seen because they used deeper or slightly more offshore habitat (e.g. Mafia Island, Tanzania [52]). If such ‘cryptic residency’ occurs near Oslob, the provisioning aggregation could be explained, at least in part, by a shift in depth use of resident sharks (i.e. increased time at the surface) as opposed to an influx of previously non-resident individuals. This is an important question to resolve because a shift in broad-scale horizontal movements would likely have more substantial ecological implications (e.g. re-distribution of whale sharks in the region) than a change in depth use among resident sharks.

### 4.2. Increasing use of the provisioning site?

While our time series is still very short, there was evidence of increasing shark abundance at the provisioning site over three years. This increase was driven specifically by an increase in the number of sharks visiting the site during approximately May–November (figure 3 and 6*b*). While this trend must be interpreted extremely cautiously, high seasonal shark abundance at the provisioning site could have important consequences for both sharks and humans. In particular, high shark densities in such a restricted space— often the majority of sharks within the interaction area are concentrated in an area not more than approximately 20 m × 50 m—could lead to elevated disturbance levels or injury arising from physical contact between sharks and people or boats [28,53]. While a code of conduct for operators at Oslob exists, including a minimum distance to be maintained between humans and sharks (2 m from the body, 5 m from the head), non-compliance with this code of conduct is extremely high (i.e. 97% in 2014 [54]). Therefore, elevated shark stress levels, and risk of injury to humans, may be significant concerns during peak season, especially if shark abundance continues to increase (see also [37]).

### 4.3. Long-term consequences of provisioning

The long-term impacts of whale shark provisioning at Oslob may be mitigated by the fact that these sharks are immature so reproductive behaviour is not immediately affected. However, provisioning of juveniles still has the potential to negatively influence whale shark populations. For example, permanent or prolonged residency at the provisioning site equates to less time spent foraging naturally and learning spatiotemporal patterns of prey distribution (e.g. predictable fish spawning [55]), which could reduce foraging efficiency, alter shark distributions and lead to dependency on provisioning in later life stages. Furthermore, it is unclear how the body condition and growth rates of sharks that consume large amounts of provisioned food (sergestid shrimp harvested hundreds of kilometres away and shipped and stored on ice for up to five days) compared to those of conspecifics that feed on a broader diet. It is also unclear how high shark densities around the provisioning site affect plankton availability for the rest of the marine community. Lastly, it is possible that sharks may increasingly associate boats with food, which could lead to increased boat strikes, entanglement in fishing gear or susceptibility to harvest where legal or illegal hunts may still occur [56,57]. However, there is currently no evidence that highly resident sharks at Oslob suffer major injuries (e.g. propeller strikes) more frequently than less resident individuals based on scarring frequencies [31] (G. Araujo 2016, unpublished data).

### 4.4. What drives variation in residency behaviour?

A key question regarding whale sharks at Oslob, and other shark species at other provisioning sites [18,22,37], is what drives diversity in site visitation patterns. Why do some individuals become highly resident while others not at all? At Oslob, with its small provisioning area, density-dependence will surely play a role because a limited number of individuals can feed efficiently at one time. In addition, the timing of site discovery appears to have been influential because many of the highly resident sharks in this study were among the first identified at the site (electronic supplementary material, figure S1). Many of these sharks adopted a vertical suction-feeding tactic wherein they position themselves in the water directly beneath a feeder boat and consume shrimp directly from the feeder [31]. These individuals may have benefitted from relatively low shark density in the early stages of provisioning and were thereby able to learn this efficient foraging tactic [54]. This may have reduced opportunities for subsequent sharks to do the same because space at feeder boats would have been limited.

A variety of other factors could also contribute to variation in whale shark residency patterns. There was a significant difference in body size between residency groups, with highly resident sharks being the smallest at the time of initial identification followed by seasonal, infrequent and single-sighting sharks. However, residency group explained a low proportion of variance in body size so size does not appear useful for predicting how an individual will behave. Alternatively, it is possible that some sharks are inherently more amenable to the conditions of the provisioning site than others. Indeed, animal personality (i.e. repeatable behavioural differences among individuals that are consistent across contexts [58]) has been demonstrated in diverse taxa but has only recently received attention in sharks [59]. Variation in the ability to optimize feeding techniques for the provisioning site (i.e. vertical feeding [54]) could also lead to among-individual differences in site visitation due to the likely higher energy intake by efficient vertical feeders. Finally, the willingness to tolerate disturbance at the provisioning site may vary with an animal's energetic state. For example, it is possible that individuals in poorer condition tolerate higher levels of disturbance to feed at the provisioning site because they have more to lose by abandoning this food source than individuals in better condition [60]. These and other drivers of individual variation in provisioning site use offer important avenues of future research.

## 5. Conclusion

The provisioning of juvenile whale sharks at Oslob, Philippines has led to the emergence of a large shark-viewing industry that brings substantial tourism revenue to a remote community but whose long-term impacts on the sharks remain poorly understood. Our research to date has shown that provisioning has conditioned sharks to associate this site with food and resulted in habituation of some individuals to disturbance [54]. In addition, provisioning appears to have modified the horizontal and/or vertical movements of a small proportion of individuals (i.e. highly resident and some seasonal sharks) over several months to years ([31], this study). Among other reasons, this is a concern because prolonged or permanent residency at the provisioning site could reduce foraging efficiency, alter shark distributions and lead to dependency on provisioning in later life stages. In comparison, the majority of sharks identified at the provisioning site over three years were only present intermittently, suggesting minimal impact of provisioning on the movements of these individuals. Critically, the impacts of provisioning on shark body condition and growth rates remain unknown and the socioeconomic costs and benefits of this operation have not yet been formally assessed. In the light of the steep decline in the whale shark population in this region [32] and the recent emergence of additional provisioning sites elsewhere in the Coral Triangle (e.g. Gorontalo, Talisayan and Cenderawasih, Indonesia), we recommend that the precautionary principle be followed pending further research into the long-term consequences of whale shark provisioning.

## Supplementary Material

Fig. S1: Individual site visitation histories of all sharks

## Supplementary Material

Sample code and data

## Data accessibility

All shark presence–absence histories are shown in the electronic supplementary material, figure S1. Sample code and data showing how we used permutation tests and PCoA to categorize shark residency patterns are also included in the electronic supplementary material. All shark sightings data can be accessed online at https://www.whaleshark.org.
