# Supplementary figures and images for: Feeding the world's largest fish: highly variable whale shark residency patterns at a provisioning site in the Philippines
Source: R Soc Open Sci. 2017 Sep 27;4(9):170394. doi: 10.1098/rsos.170394 (PMC5627090; doi:10.1098/rsos.170394)

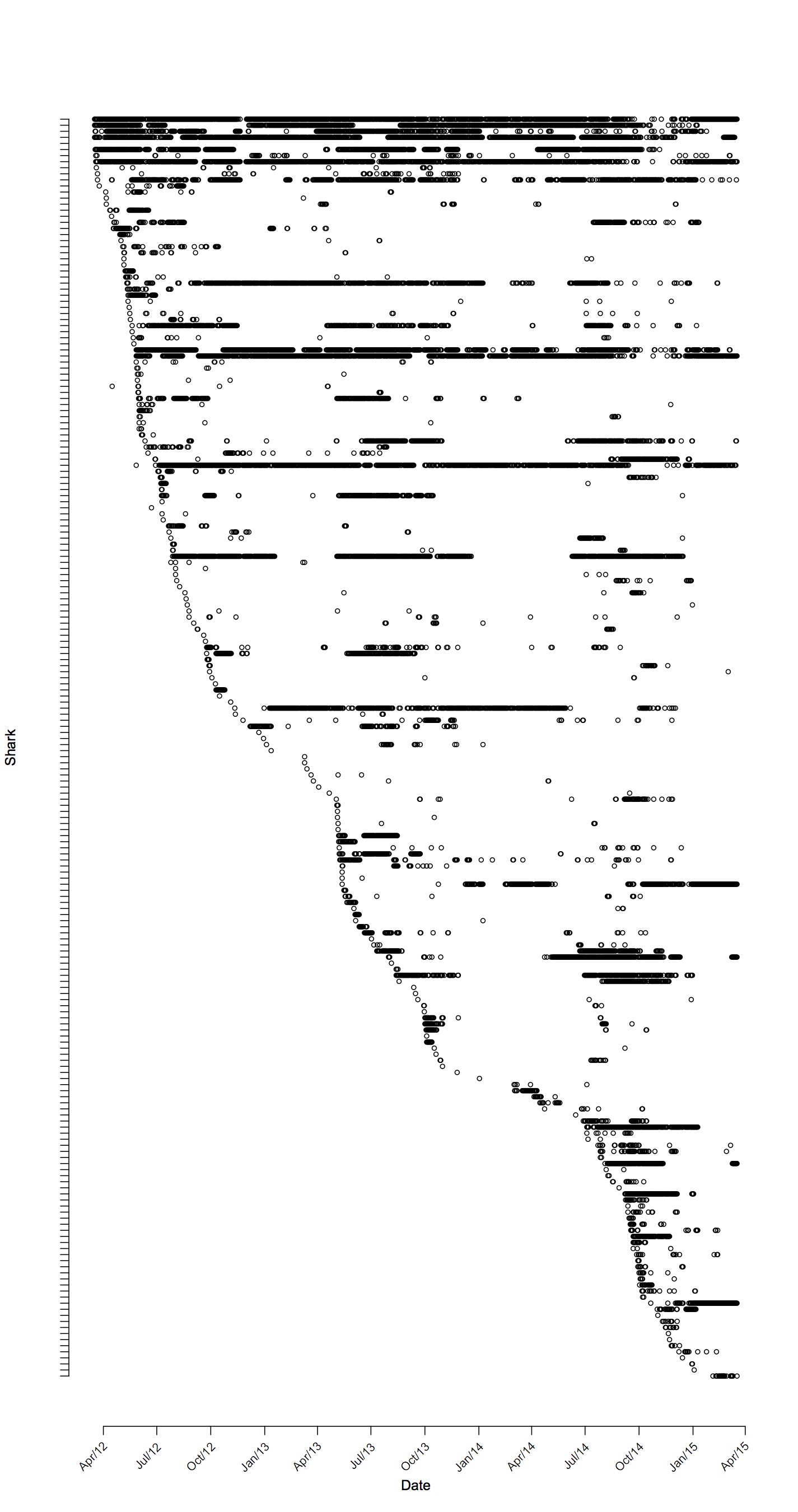

Supplement: Fig. S1: Individual site visitation histories of all sharks [file rsos170394supp1.jpg]
